# GABAergic neurons in the olfactory cortex projecting to the lateral hypothalamus in mice

**DOI:** 10.1038/s41598-019-43580-1

**Published:** 2019-05-09

**Authors:** Koshi Murata, Tomoki Kinoshita, Yugo Fukazawa, Kenta Kobayashi, Kazuto Kobayashi, Kazunari Miyamichi, Hiroyuki Okuno, Haruhiko Bito, Yoshio Sakurai, Masahiro Yamaguchi, Kensaku Mori, Hiroyuki Manabe

**Affiliations:** 1 Division of Brain Structure and Function, Faculty of Medical Sciences, University of Fukui, Fukui, 910-1193 Japan; 2 Life Science Innovation Center, Faculty of Medical Science, University of Fukui, Fukui, 910-1193 Japan; 3 Laboratory of Neural Information, Graduate School of Brain Science, Doshisha University, Kyoto, 610-0394 Japan; 4 Research Center for Child Mental Health Development, Faculty of Medical Sciences, University of Fukui, Fukui, 910-1193 Japan; 5 Section of Viral Vector Development, National Institute for Physiological Sciences, Aichi, 444-8585 Japan; 6 Department of Molecular Genetics, Institute of Biomedical Sciences, Fukushima Medical University School of Medicine, Fukushima, 960-1295 Japan; 7 Laboratory for Comparative Connectomics, RIKEN Centre for Biosystems Dynamics Research, Hyogo, 650-0047 Japan; 8 Department of Biochemistry and Molecular Biology, Kagoshima University Graduate School of Medical and Dental Sciences, Kagoshima, 890-8544 Japan; 9 Department of Neurochemistry, Graduate School of Medicine, The University of Tokyo, Tokyo, 113-0033 Japan; 10 Department of Physiology, Kochi Medical School, Kochi University, Kochi, 783-8505 Japan; 11 Department of Physiology, Graduate School of Medicine, The University of Tokyo, Tokyo, 113-0033 Japan

**Keywords:** Neural circuits, Olfactory cortex

## Abstract

Olfaction guides goal-directed behaviours including feeding. To investigate how central olfactory neural circuits control feeding behaviour in mice, we performed retrograde tracing from the lateral hypothalamus (LH), an important feeding centre. We observed a cluster of retrogradely labelled cells distributed in the posteroventral region of the olfactory peduncle. Histochemical analyses revealed that the majority of these retrogradely labelled projection neurons expressed glutamic acid decarboxylase 65/67 (GAD65/67), but not vesicular glutamate transporter 1 (VGluT1). We named this region containing GABAergic projection neurons the ventral olfactory nucleus (VON) to differentiate it from the conventional olfactory peduncle. VON neurons were less immunoreactive for DARPP-32, a striatal neuron marker, compared to neurons in the olfactory tubercle and nucleus accumbens, which distinguished the VON from the ventral striatum. Fluorescent labelling confirmed putative synaptic contacts between VON neurons and olfactory bulb projection neurons. Rabies-virus-mediated trans-synaptic labelling revealed that VON neurons received synaptic inputs from the olfactory bulb, other olfactory cortices, horizontal limb of the diagonal band, and prefrontal cortex. Collectively, these results identify novel GABAergic projection neurons in the olfactory cortex that may integrate olfactory sensory and top-down inputs and send inhibitory output to the LH, which may modulate odour-guided LH-related behaviours.

## Introduction

The central olfactory system translates odour information into motivated behaviours, including appetite-based food approach and eating behaviours^1^. Recent studies have revealed neuronal circuit mechanisms by which odorants evoke specific behaviours, such as fear responses to predator odours^2,3^ and attractive responses to social odours^4^. However, it is still unclear how central olfactory neural circuits control feeding-related behaviours in mammals.

Odorants activate olfactory sensory neurons and are coded by activation of specific combinations of glomeruli in the olfactory bulb (OB), the first relay centre of the central olfactory system^5^. Mitral cells and tufted cells (M/TCs) are projection neurons in the OB. They convey odour information to several areas in the olfactory cortex which is composed of the anterior olfactory nucleus (AON), tenia tecta, dorsal peduncular cortex, anterior piriform cortex (APC), olfactory tubercle (OT), posterior piriform cortex (PPC), cortical amygdaloid nuclei, and lateral entorhinal cortex^6^.

Knowledge of neural pathways from the olfactory cortex to the lateral hypothalamus (LH), an important feeding centre, is crucial to identify how olfactory information is translated into feeding-related behaviours^7^. Price *et al*. examined the neural connections between the central olfactory system and LH in rats^8^, and reported that several areas of the olfactory cortex have axonal projections to the LH. Since then, however, there have been a paucity of neuroanatomical studies investigating how olfactory information is conveyed to the LH in mammals.

Here, we examined the neural pathways from the central olfactory system to the LH in mice using cholera toxin B subunit (CTB) alongside viral and genetic techniques to trace neural circuits. In agreement with previous findings indicating that the LH receives inputs from the AON, ventral tenia tecta (VTT), APC, and OT^8^, we observed a subset of retrogradely labelled cells clustered in a postero-ventral region of the olfactory peduncle. Our analyses revealed that the majority of LH-projecting neurons in this region were GABAergic, in contrast to the AON and VTT where principal neurons are glutamatergic. We also observed that these GABAergic neurons extended dendrites to layer I of the olfactory cortex and received synaptic inputs from olfactory bulb neurons. These results suggest a novel population of GABAergic neurons in the olfactory cortex projecting to the LH.

## Results

### Retrograde tracing from the LH revealed a cluster of GABAergic neurons in the olfactory peduncle

**Figure 1:**
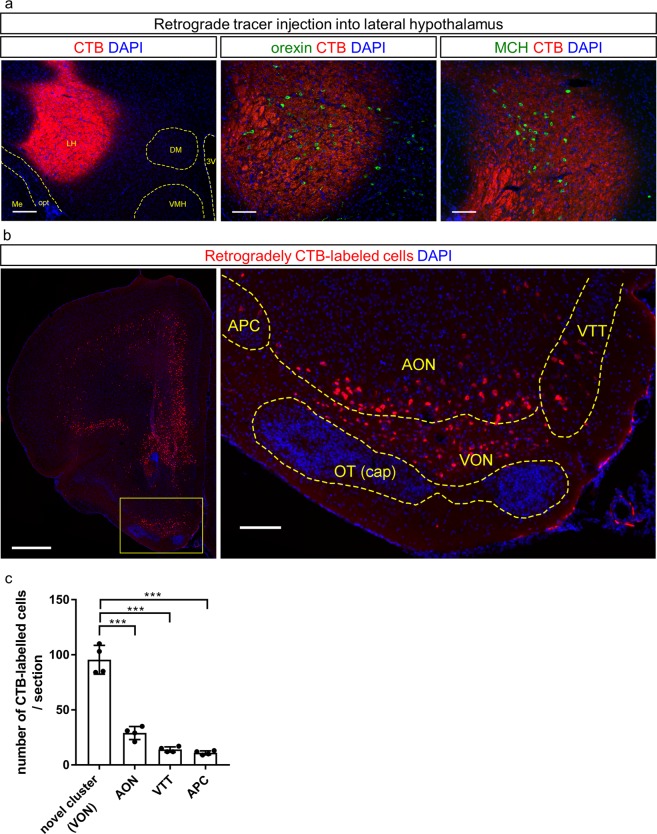
Retrograde tracing revealed a cluster of neurons projecting to the lateral hypothalamus (LH) surrounded by the olfactory cortex. (**a**) Coronal sections of the LH after injection of Alexa 555-conjugated cholera toxin B subunit (CTB, red) with DAPI staining (blue). Immunostaining for orexin (middle, green) or melanin-concentrating hormone (MCH) (right, green) was performed. Scale bars: 200 μm in left panel, 100 μm in middle and right panels. LH, lateral hypothalamus; DM, dorsomedial hypothalamus; VMH ventromedial hypothalamus, 3V, third ventricle; Me, medial amygdaloid nucleus; opt, optic tract. The structure boundaries were drawn using the Franklin and Paxinos mouse brain atlas^31^. (**b**) CTB-labelled cells in the frontal cortex and olfactory cortex. The inset in the left panel is magnified in the right panel. Scale bars: 500 μm in left panel, 100 μm in right panel. (**c**) The average number of CTB-labelled cells in each olfactory cortical area (average of three coronal sections) Data are shown as average ± SD with individual plots. ***p < 0.001. APC, anterior piriform cortex; AON, anterior olfactory nucleus; VTT, ventral tenia tecta; VON, ventral olfactory nucleus; OT, olfactory tubercle.

**Figure 2:**
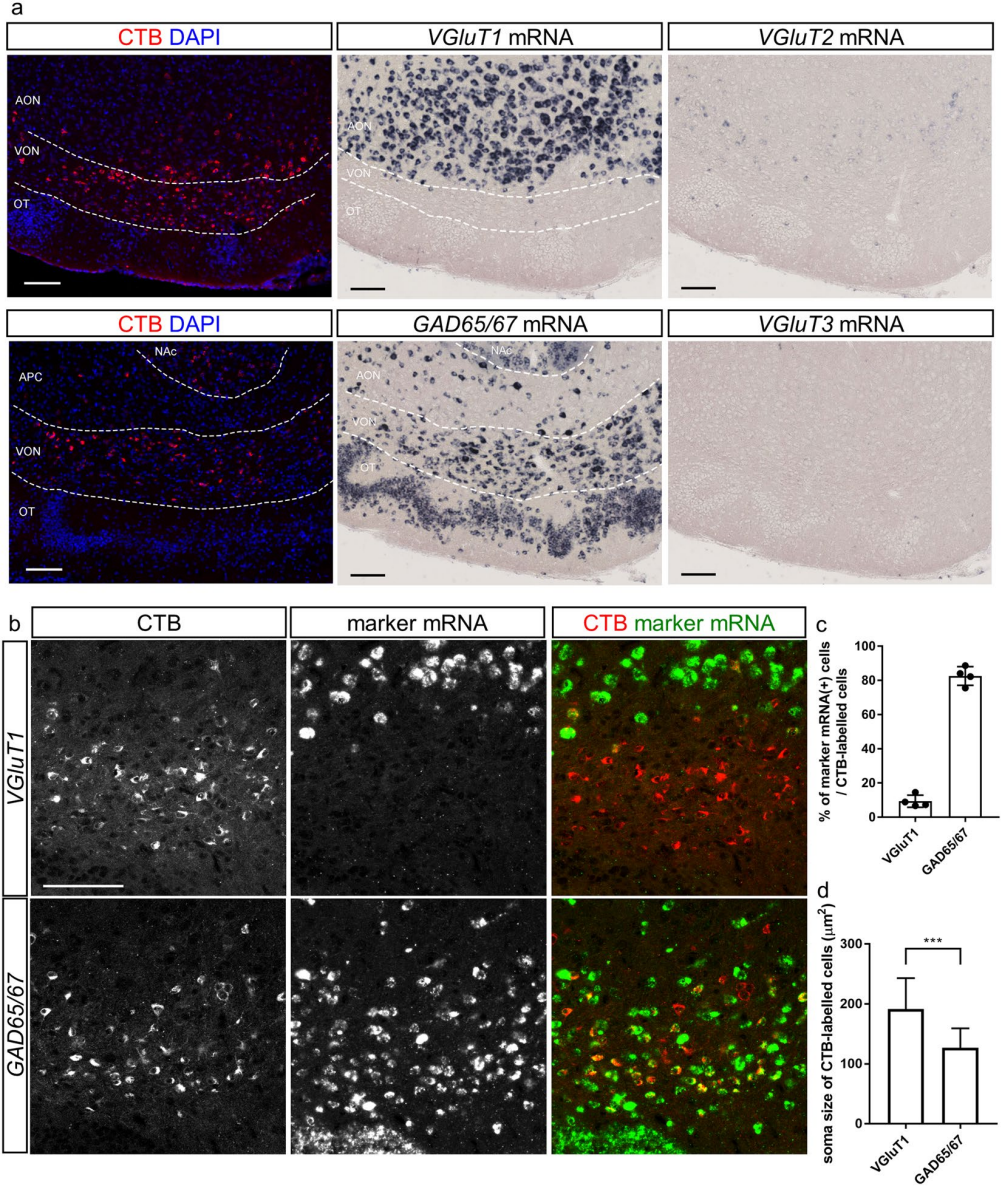
Majority of CTB-labelled neurons in the ventral olfactory nucleus (VON) are GABAergic. (**a**) *In situ* hybridization for *VGluT*s and *GAD65/67* mRNAs alongside adjacent slice images of cholera toxin B subunit (CTB)-labelled ventral olfactory nucleus (VON) neurons. Scale bars: 100 μm. AON, anterior olfactory nucleus; VON, ventral olfactory nucleus; OT, olfactory tubercle. (**b**) Double fluorescent labelling of CTB and *VGluT1* (upper panels) or *GAD65/67* (lower panels) mRNAs. Scale bar: 100 μm. (**c**) Percentage of *VGluT1* or *GAD65/67* mRNA(+) cells among CTB-labelled cells in the VON. Data are shown as average ± SD with individual data plots. (**d**) Soma size of *VGluT1* or *GAD65/67* mRNA(+) CTB-labelled cells in the VON. Data are shown as average ± SD. ***p < 0.001.

**Figure 3:**
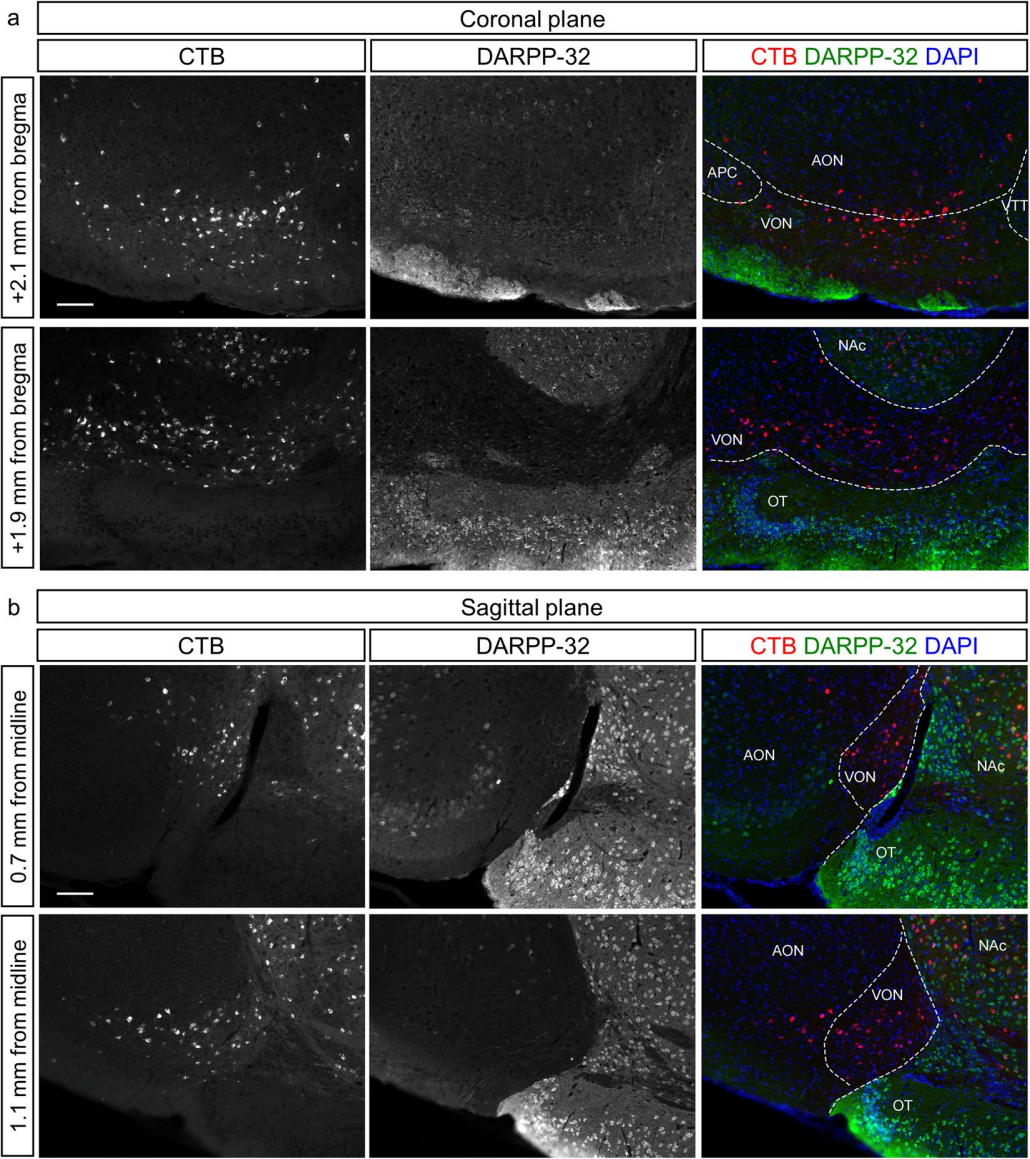
The VON is distinguishable from the ventral striatum by DARPP-32 immunoreactivity. (**a**,**b**) Coronal sections (**a**) and sagittal sections (**b**) of the VON after injection of Alexa 555-conjugated cholera toxin B subunit (CTB) (red) into the lateral hypothalamus (LH) with immunostaining for DARPP-32 (green) and DAPI staining (blue). AON, anterior olfactory nucleus; VON, ventral olfactory nucleus; OT, olfactory tubercle; NAc, nucleus accumbens. Scale bars: 100 μm.

To examine neural pathways from the olfactory cortex to the LH, we injected a retrograde tracer, CTB conjugated with Alexa 555, into the mouse LH (n = 10 mice). We targeted an area of the LH containing orexin neurons and melanin-concentrating hormone (MCH) neurons (Fig. 1a), as both these neuronal subpopulations are involved in feeding behaviours^9,10^. CTB-labelled cells were widely distributed in the brain including the prefrontal cortex (Fig. 1b, left). In accordance with previous findings demonstrating that the olfactory cortex sends axonal projections to the LH^8^, we observed CTB-labelled cells distributed in the AON, APC, and VTT (Fig. 1b, right) as well as the posterior part of the OT (data not shown). In addition to these areas, a cluster of CTB-labelled cells was noted in an area surrounded by the AON, APC, and VTT (Fig. 1b, right). All tracer-injected mice showed spread of CTB in the LH and CTB-labelled cells in the posteroventral olfactory peduncle as shown in Fig. 1b (right). Among the 10 tracer-injected mice, we selected four for subsequent analyses (Figs 1–3 and Supplementary Figs 1–3), which showed spread of CTB in the injection site was confined within the LH and did not extend to other subregions of the hypothalamus, thalamus, or amygdala (Supplementary Fig. 1). We compared the number of CTB-labelled cells in the cluster to that in surrounding olfactory cortical areas (AON, VTT, and APC). Significantly more CTB-labelled cells were observed in the cluster (96 ± 11 cells/coronal section, n = 4 mice, one-way ANOVA with post-hoc Tukey’s test, *F*_(3,12)_ = 11.26, *p* = 0.0008) than in other areas (29 ± 5 cells/section, AON; 14 ± 2 cells/section, VTT; 11 ± 2 cells/section, APC, n = 4 mice).

The size of CTB-labelled somata in the cluster appeared smaller than that of CTB-labelled somata in the posterior part of the AON (Fig. 2b,d), raising the possibility that they were of a different neuronal subtype to that of principal neurons in the AON. Projection neurons in the olfactory cortex, except for the OT, are thought to be glutamatergic^6^. To examine whether the CTB-labelled cells were glutamatergic or GABAergic, we performed *in situ* hybridization for mRNA of vesicular glutamate transporters (VGluTs) and glutamic acid decarboxylase (GAD) 65/67 in this area (Fig. 2). *VGluT1*-expressing cells were distributed in the dorsal edge of the area in which CTB-labelled cells were clustered. We observed a cluster of *GAD65/67*-expressing cells just above the rostral tip of the OT, which was characterized by the cap region including GABAergic dwarf cells^11^; this overlapped with the distribution of CTB-labelled cells. *VGluT2*- and *VGluT3*-expressing cells were scarcely observed in this area. We then directly examined the expression of *VGluT1* and *GAD65/67* mRNAs in CTB-labelled cells by double fluorescent labelling (Fig. 2b). *VGluT1*-expressing CTB-labelled cells were distributed in the ventral border of the AON, and 9.3 ± 3.6% of the CTB-labelled cells were *VGluT1*-positive (n = 4 mice, total 404 cells). In contrast, 82.6 ± 5.4% of the CTB-labelled cells were *GAD65/67*-positive (n = 4 mice, total 418 cells), suggesting that principal neurons in this area were GABAergic. Specificity of fluorescent labelling for *VGluT1* and *GAD65/67* mRNAs was confirmed by examining CTB-labelled cells in the medial prefrontal cortex, which demonstrated that 87.5 ± 0.7% of retrogradely labelled cells were *VGluT1*(+) (n = 3 mice, total 394 cells) and 3.2 ± 1.2% were *GAD65/67*(+) (n = 3 mice, total 349 cells) (Supplementary Fig. 2). Soma size of *GAD65/67*(+) CTB-labelled cells was significantly smaller than that of *VGluT1*(+) CTB-labelled cells (121 ± 27 μm^2^ vs. 191 ± 52 μm^2^, n = 40 cells and 36 cells, respectively, *t*_74_ = 6.605; unpaired *t*-test, Fig. 2d). These cellular profiles suggest that the GABAergic CTB-labelled cells did not belong to the conventional olfactory peduncle, and confirmed previous reports that a subset of glutamatergic neurons in the posteroventral AON sends axonal projections to the LH^8^. We therefore termed this area the ventral olfactory nucleus (VON) as the cluster of GABAergic neurons projecting to the LH was located just ventral to the posterior part of the AON.

One possible cellular profile of GABAergic neurons in the VON is medium spiny neurons in the OT and nucleus accumbens (NAc); namely, the ventral striatum^12,13^. To examine whether CTB-labelled cells in the VON were different to medium spiny neurons of the ventral striatum, we performed immunostaining for DARPP-32, a marker for striatal neurons^14^, alongside CTB labelling (Fig. 3 and Supplementary Fig. 3). CTB-labelled cells were distributed in regions that demonstrated either strong or weak DARPP-32 immunoreactivity, corresponding to the ventral striatum and VON, respectively. These results implied that GABAergic neurons in the VON were indeed distinct cellular populations to medium spiny neurons in the OT and NAc.

### Rabies virus-mediated trans-synaptic retrograde labelling from the LH to the VON

**Figure 4:**
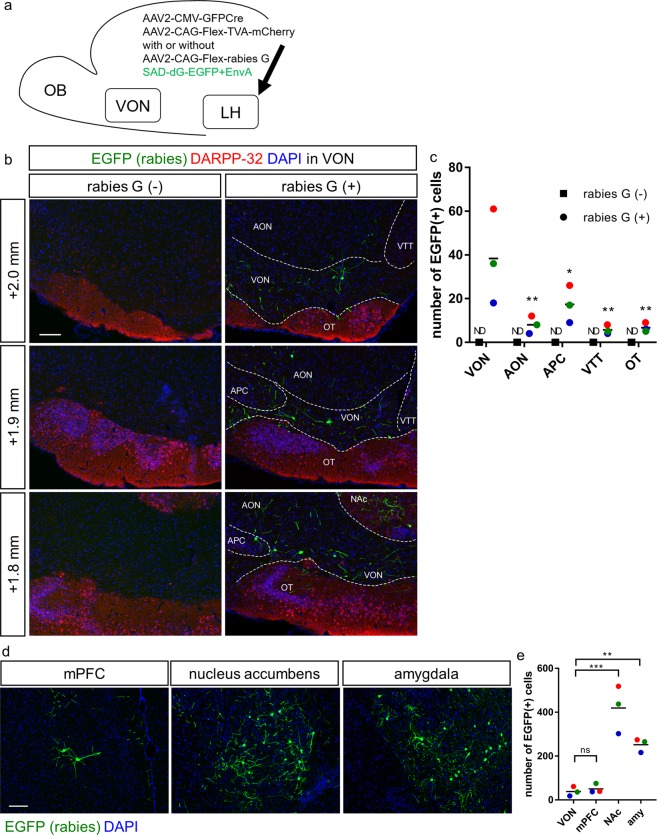
Trans-synaptic retrograde spread of rabies virus from the LH to the VON. (**a**) Schema of virus-mediated retrograde tracing. We first injected a mixture of adeno-associated viruses (AAVs) encoding CMV-GFPCre and CAG-Flex-TVA-mCherry either with or without CAG-Flex-rabies G into the lateral hypothalamus (LH). Two weeks later, SAD-dG-EGFP-EnvA was injected into the LH. We examined the distribution of EGFP-labelled presynaptic cells in the ventral olfactory nucleus (VON). (**b**) EGFP-labelled presynaptic neurons (green) were observed in the VON when rabies G-encoding AAV was concomitantly injected (right panels). Immunostaining for DARPP-32 (red) was used to discriminate the VON from the olfactory tubercle (OT) and nucleus accumbens (NAc). AON, anterior olfactory nucleus; VON, ventral olfactory nucleus; APC, anterior piriform cortex; OT, olfactory tubercle; NAc, nucleus accumbens. Scale bar: 100 μm. (**c**) The number of EGFP-labelled cells in the olfactory cortical areas. Data are shown as mean with individual plots. Statistical differences were tested between data of rabies G(+) in the VON vs. data of rabies G(+) in other areas by two-way ANOVA with post-hoc Tukey’s test. ND, not detected; *p < 0.05; **p < 0.01. (**d**) EGFP-labelled presynaptic neurons (green) in the mPFC (left), NAc (middle), and amygdala (right). Scale bar: 100 μm. (**e**) The number of EGFP-labelled cells in the VON, mPFC, NAc, and amygdala. Data for the VON are similar to the data in (**c**). Data are shown as mean with individual plots. Each colour represents the data from one mouse. ns, not significant; **p < 0.01; ***p < 0.001.

CTB labelling did not unequivocally indicate whether the labelled cells had synaptic connections to the postsynaptic neurons or whether their axon fibres were passing through the injection site^15^. To address whether VON neurons formed synaptic contacts in the LH, we performed trans-synaptic labelling of LH neurons combining an EnvA-pseudotyped glycoprotein-deleted rabies virus encoding EGFP (SAD-dG-EGFP + EnvA) with AAV-mediated expression of the EnvA receptor (TVA) and rabies glycoprotein^16–19^. This labelling revealed input pathways to LH neurons from widespread brain regions. We injected Cre-encoding AAV and Cre-dependent AAVs encoding TVA-mCherry and rabies glycoprotein into the LH, followed by injection of the modified rabies virus (SAD-dG-EGFP + EnvA) into the LH^16–19^ (n = 9 mice, Fig. 4a). Because trans-synaptic spread of the rabies virus is mediated by the rabies glycoprotein^19^, we compared efficacy of retrograde labelling with and without rabies G expression in the LH in four mice. Coronal brain sections of 20 μm thickness were prepared from the anterior tip of the OB through the entire brain, and every fifth section was analysed. Starter cells of TVA-mCherry(+) and EGFP(+) cells were observed in the LH in cases with and without rabies G-coding AAV (data not shown). Presynaptic cells of cytosolic EGFP(+) TVA-mCherry(−) cells were observed in the VON when we concomitantly injected rabies G-coding AAV into the LH (Fig. 4b, right panels and 4c). In contrast, cytosolic EGFP(+) TVA-mCherry(−) cells were never observed in the VON or other brain regions in the absence of rabies G-coding AAV in the LH (Fig. 4b, left panels and 4c). We then quantified the number of cytosolic EGFP(+) TVA-mCherry(−) cells (presynaptic cells) in three mice that showed localized distribution of starter cells in the LH among the nine mice that received rabies G-encoding AAV. Among the olfactory cortical areas, there were the most presynaptic cells (EGFP(+) cells) in the VON (VON, 38 ± 18 cells; AON, 8 ± 3 cells; APC, 17 ± 7 cells; VTT, 6 ± 2 cells; OT, 7 ± 2 cells for rabies G(+) mice, n = 3 mice; no EGFP(+) cells for rabies G(−) mice, n = 4 mice; two-way ANOVA with post-hoc Tukey’s test) (Fig. 4c). We then compared the number of presynaptic neurons from the VON to the LH with that from three other major presynaptic regions: the medial prefrontal cortex, NAc, and amygdala^20,21^. The number of EGFP(+) cells in the VON was significantly less than that in the NAc and amygdala (Fig. 4e), and as large as that in the medial prefrontal cortex. The results of rabies glycoprotein-dependent retrograde spread of rabies virus support the concept that neurons in the VON form synaptic contacts in the LH, although the number of synaptic inputs from the VON was less than from the NAc and amygdala.

### Putative synaptic contacts from OB M/TCs onto VON neurons

**Figure 5:**
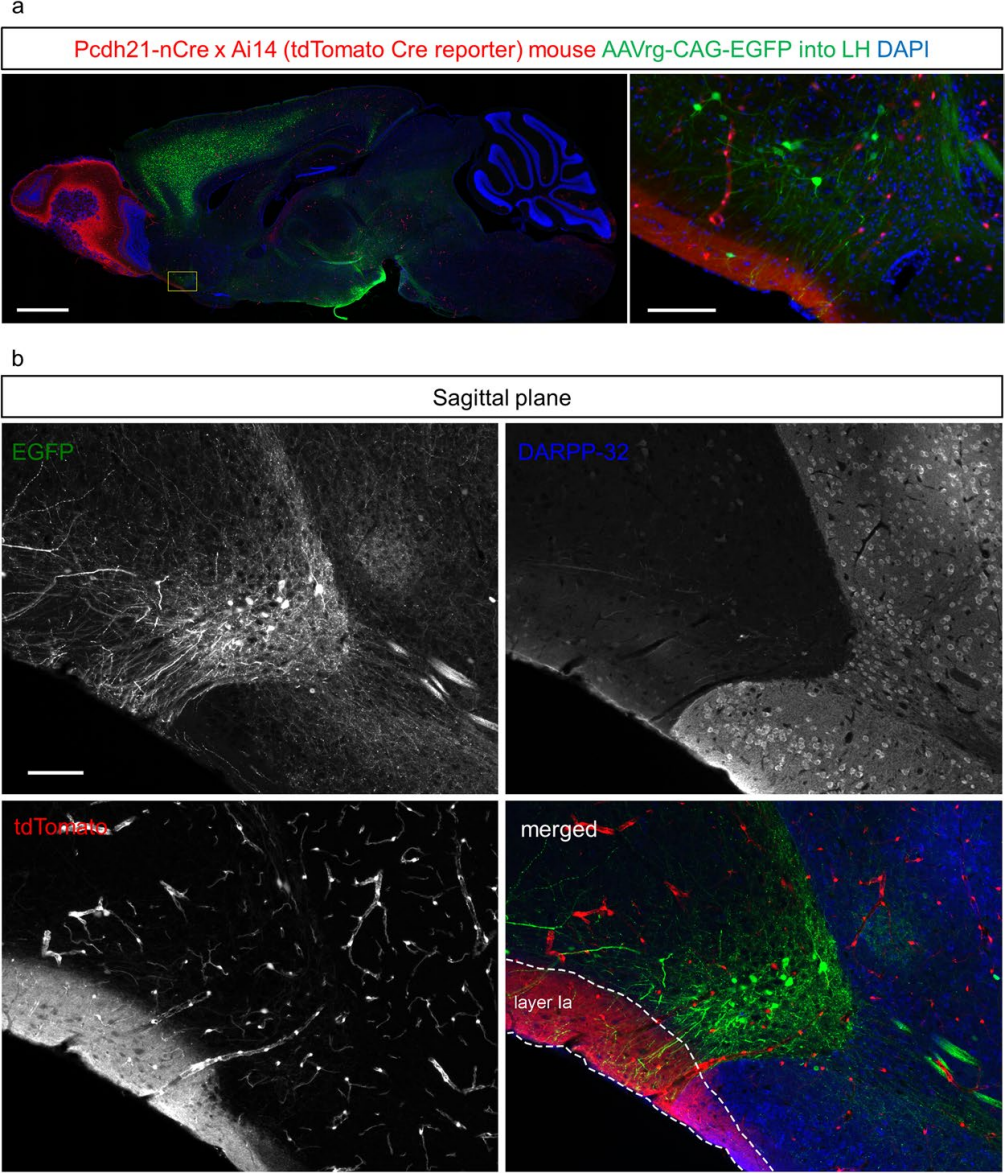
Mitral and tufted cells in the olfactory bulb send axonal projections to the VON. (**a**) Sagittal sections of the whole brain of mitral and tufted cells (M/TCs) in a tdTomato-labelled transgenic mouse after injection of retrograde adeno-associated virus (AAV) vector encoding EGFP into the lateral hypothalamus (LH). The inset of the left panel is magnified in the right panel. Note that vascular endothelial cells and a subset of neurons in the olfactory cortex were also labelled with tdTomato. Scale bars: 1 mm in left panel, 100 μm in right panel. (**b**) Sagittal sections of the ventral olfactory nucleus (VON) with immunostaining for DARPP-32 (blue). Dendrites of EGFP-labelled VON neurons (green) innervated layer Ia of the olfactory cortex which was innervated by axons of tdTomato-labelled M/TCs (red). Scale bar: 100 μm.

**Figure 6:**
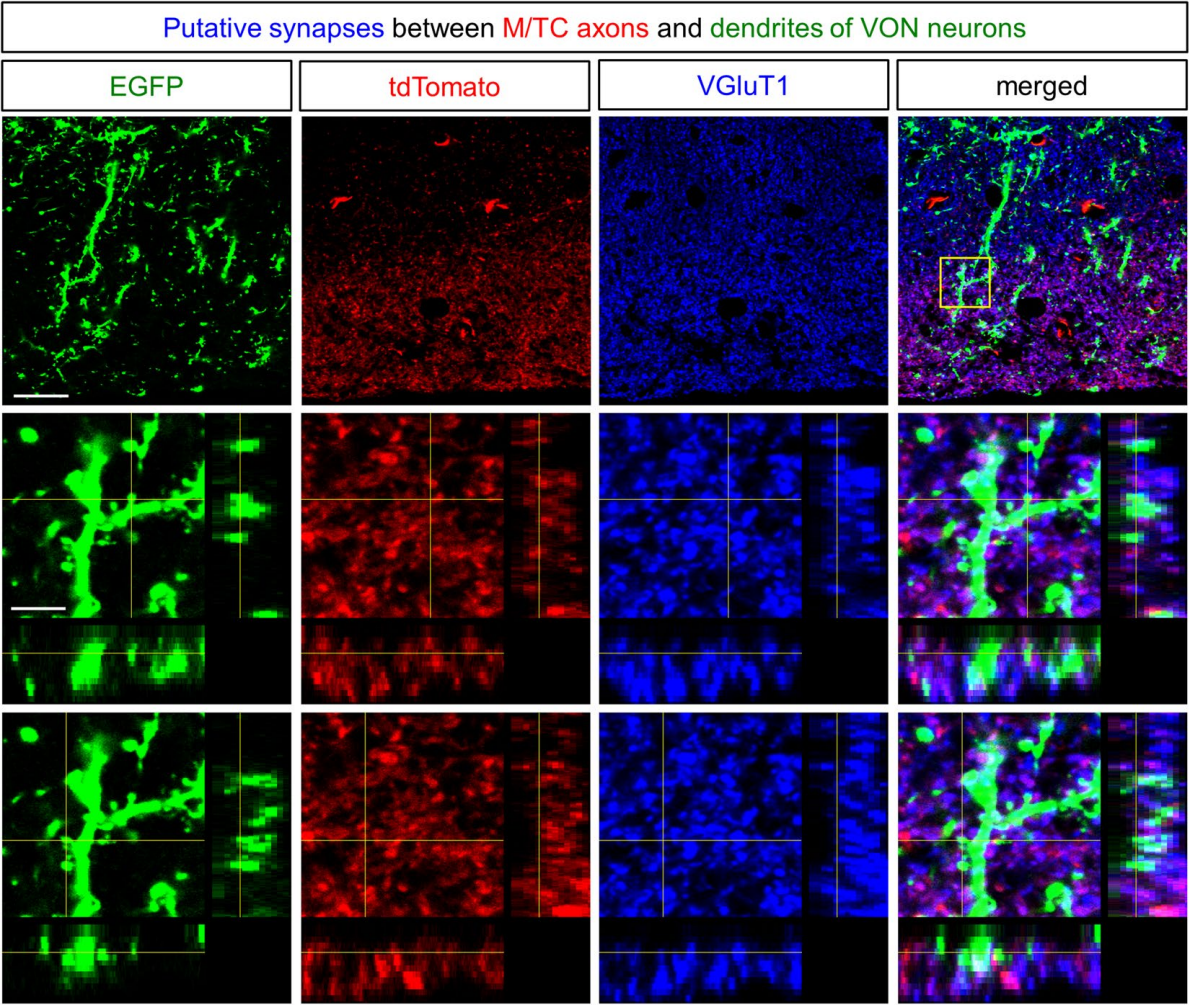
Synaptic contacts between mitral and tufted cells and dendritic spines of VON neurons. Upper four panels depict single plane confocal images of dendrites of EGFP-labelled ventral olfactory nucleus (VON) neurons (green), axons of tdTomato-labelled mitral and tufted cells (M/TCs) (red), and immunoreactivity for vesicular glutamate transporter 1 (VGluT1) (blue) in layer Ia of the VON. Lower eight panels are magnified views of the inset with XZ-YZ views. Scale bars: 20 μm for upper four panels, 5 μm for lower eight panels.

One of the criteria of the olfactory cortex specifies areas which receive direct synaptic inputs from M/TCs, projection neurons in the OB^6^. To investigate putative synaptic contacts from M/TCs onto the dendrites of VON neurons, we used a transgenic mouse line in which M/TCs were labelled by tdTomato (Pcdh21-nCre x tdTomato Cre reporter line (Ai14)^22,23^). In this transgenic mouse line, vascular endothelial cells, a subset of neurons in the olfactory cortex, and M/TCs were labelled by tdTomato (Fig. 5). We injected retrograde adeno-associated virus (AAV) encoding EGFP (AAVrg-CAG-EGFP^24^) into the LH of Pcdh21-nCre x tdTomato Cre mice (n = 7, Figs 5 and 6). In all the AAV-injected mice, we observed that EGFP-labelled dendrites of VON neurons received innervation by tdTomato-labelled axons of M/TCs in layer Ia of the olfactory cortex (Fig. 5 and Supplementary Fig. 4). Notably, dendrites of VON neurons were excluded from regions with strong DARPP-32 immunoreactivity; namely, the NAc and OT (Fig. 5b and Supplementary Fig. 4).

We next performed immunostaining for VGluT1, a presynaptic marker of M/TCs^25,26^. We examined apposition of VGluT1 signals and dendrites of VON neurons (Fig. 6). EGFP-labelled dendrites of VON neurons possessed spines (Figs 5b and 6). Axonal boutons of tdTomato-labelled M/TCs in layer Ia were apposed to dendritic spines of VON neurons, which colocalised with VGluT1 immunoreactivity (Fig. 6, lower eight panels). These results suggested that M/TCs provided putative synaptic contacts onto VON neurons.

### Trans-synaptic retrograde tracing from the VON using a modified rabies virus

**Figure 7:**
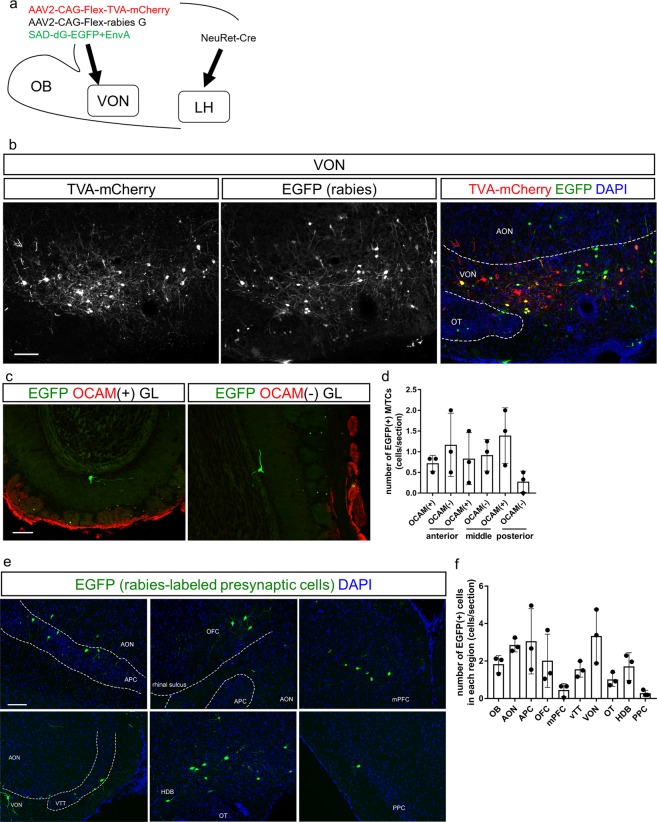
Rabies virus-mediated trans-synaptic retrograde tracing from the VON. (**a**) Schema of virus-mediated neuronal pathway-specific retrograde tracing. We first injected a retrograde lentiviral vector encoding Cre (NeuRet-Cre) into the lateral hypothalamus (LH), and Cre-dependent adeno-associated virus (AAV) vector TVA-mCherry and rabies G into the VON. Two weeks after the first injection, EnvA-pseudotyped glycoprotein-deleted rabies virus encoding EGFP was injected into the VON. We then examined the distribution of EGFP-labelled presynaptic cells throughout the whole brain. (**b**) Coronal sections of the VON. Left, TVA-mCherry expressing neurons; middle, rabies-derived EGFP expressing neurons; right, colour merged with DAPI staining. Yellow cells indicate TVA-mCherry(+) EGFP(+) starter cells. AON, anterior olfactory nucleus; VON, ventral olfactory nucleus; OT, olfactory tubercle. Scale bar: 100 μm. (**c**) EGFP-labelled presynaptic cells in the OB. (**d**) The number of EGFP-labelled M/TCs in the OB. Data are shown as mean ± SD with individual plots. (**e**) Distribution of EGFP-labelled presynaptic cells projecting to the VON. AON, anterior olfactory nucleus; APC, anterior piriform cortex; OFC, orbitofrontal cortex; mPFC, medial prefrontal cortex; HDB, horizontal limb of the diagonal band; OT, olfactory tubercle; PPC, posterior piriform cortex. Scale bar: 100 μm. (**f**) The percentage of the number of EGFP-labelled cells in each region over total number of EGFP-labelled cells across the whole brain. Data are shown as mean ± SD with individual plots.

To further confirm whether VON neurons received direct synaptic inputs from M/TCs in the OB, we performed trans-synaptic labelling of VON neurons combining an EnvA-pseudotyped glycoprotein-deleted rabies virus encoding EGFP (SAD-dG-EGFP + EnvA) with AAV-mediated expression of the EnvA receptor (TVA) and rabies glycoprotein^16–19^. To achieve selective initial infection of VON neurons by the rabies virus, we used the neural pathway specific-tracing method (tracing the relationship between input and output, TRIO^27^). We first injected a retrograde Cre-encoding lentiviral vector (NeuRet-Cre^28^) into the LH, and Cre-dependent AAV vectors encoding TVA-mCherry and rabies G into the VON. Two weeks after injections, we injected the modified rabies virus (SAD-dG-EGFP + EnvA) into the VON (Fig. 7a).

In total, 11 mice were used for these tracing studies. Coronal brain sections at 20 mm thickness were prepared from the anterior tip of the OB through the whole brain, and every fifth section was analysed. Among the 11 mice, starter cells of TVA-mCherry(+) and EGFP(+) cells were localized in the VON in three mice (Fig. 7b), which were used for quantitative analysis (Fig. 7d,f). We detected EGFP-labelled cells in the OB (Fig. 7c,d), confirming that VON neurons received direct synaptic input from the OB. To evaluate the distribution of EGFP-labelled M/TCs in the OB, we divided the OB into anterior (without accessory OB, six coronal sections), middle (including accessory OB without prefrontal cortex, four coronal sections), and posterior (including prefrontal cortex, six coronal sections) areas, as well as ventral OB marker OCAM-immunopositive and OCAM-immunonegative areas^29^ (Fig. 7c). EGFP-labelled M/TCs were distributed in all six areas of the OB. The number of M/TCs was not significantly different among the six subregions (anterior OCAM(+), 0.7 ± 2 cells/section; anterior OCAM(−), 1.2 ± 0.6 cells/section; middle OCAM(+) 0.8 ± 0.5 cells/section; middle OCAM(−), 0.9 ± 0.3 cells/section; posterior OCAM(+), 1.4 ± 0.6 cells/section; posterior OCAM(−), 0.3 ± 0.2 cells/section from n = 3 mice) (Fig. 7d), suggesting that the VON received axonal inputs from areas throughout the OB. In addition to the OB (1.8 ± 0.4 cells/section), EGFP-labelled cells were also distributed in the VON (3.3 ± 1.2 cells/section), AON (2.9 ± 0.3 cells/section), OT (1.0 ± 0.3 cells/section) (Fig. 7b,e), APC (3.1 ± 1.4 cells/section) (Fig. 7e, upper left panel), orbitofrontal cortex (2.0 ± 1.2 cells/section) (Fig. 7e, upper middle panel), medial prefrontal cortex (0.5 ± 0.3 cells/section) (Fig. 7e, upper right panel), VTT (1.6 ± 0.3 cells/section) (Fig. 7e lower left panel), horizontal limb of the diagonal band (1.7 ± 0.6 cells/section) (Fig. 7e, lower middle panel), and PPC (0.3 ± 0.1 cells/section) (Fig. 7e, lower right panel) (n = 3 mice). These results support that the VON meets the criterion of the olfactory cortex that receives direct synaptic inputs from M/TCs, and further suggest that, in addition to afferent input from the OB, the VON receives inputs from various areas of the olfactory cortex, a part of the diagonal band, and the prefrontal cortex (Fig. 7f).

## Discussion

In this study, we examined the neural pathways from the olfactory cortex to the LH in mice using retrograde tracing from the LH. We observed a group of retrogradely labelled cells clustered in a postero-ventral region of the olfactory peduncle. Our neuroanatomical and histochemical analyses revealed that this region predominantly comprised GABAergic neurons, thereby distinguishing it from the AON and VTT in which the principal neurons are glutamatergic. Furthermore, this region received synaptic inputs from M/TCs as well as other olfactory cortices, and horizontal limb of the diagonal band, and prefrontal cortex. According to Price, Neville, and Haberly, one of the criteria for the olfactory cortex is “those areas that receive direct synaptic input from the olfactory bulb^6,30^”. These results suggest a novel population of GABAergic neurons in the olfactory cortex that integrate olfactory sensory and top-down inputs and project to the LH.

In accordance with the previous report by Price *et al*. in rats^8^, we observed retrogradely labelled cells in the olfactory cortical areas (AON, VTT, APC, and OT) using retrograde tracing from the LH in mice (Figs 1 and 4c). In addition to these areas, we identified a cluster of retrogradely-labelled cells in a posterior part of the olfactory peduncle surrounded by the AON, VTT, APC, and OT (Figs 1b and 3). It is unclear whether the Price study^8^ observed a cluster of retrogradely labelled cells in the posterior olfactory peduncle (what we have termed the “VON”) or attributed the cluster to other olfactory cortical areas such as the AON and VTT. The different species studied or sensitivity of tracers may contribute to the discrepancy between the report by Price *et al*. and our current results. Our histochemical analyses revealed that the majority of neurons in this region were GABAergic, indicating that retrogradely-labelled GABAergic projection neurons were distinct from glutamatergic projection neurons in the AON and VTT. We therefore termed this region containing GABAergic projection neurons the ventral olfactory nucleus (VON) which was located just beneath the AON. We subsequently examined the neuroanatomical features of the VON.

It may be possible that VON neurons are anteriorly displaced GABAergic medium spiny neurons of the OT and NAc as the location of the VON is just above the anterior tip of the OT and anterior to the NAc (Fig. 3). To examine this possibility, we performed immunostaining for DARPP-32 and observed that the strongly immunoreactive OT and NAc neurons were clearly distinguishable from the less immunoreactive VON neurons (Figs 3 and 5, and Supplementary Figs 3 and 4). The cytoarchitecture of the VON is different from other olfactory cortices in that it lacks glutamatergic pyramidal cells or DARPP-32-expressing striatal neurons, suggesting that developmental origin of the VON may also be different from other olfactory cortices. The region of distribution of VON neurons was absent from the olfactory cortical areas depicted in the mouse brain atlas by Paxinos and Franklin^31^ (Supplementary Fig. 3).

Fluorescent labelling revealed putative synaptic contacts between M/TCs in the OB and VON neurons (Figs 5 and 6). The dendrites of VON neurons projected in an antero-ventral direction toward the cortical surface and extended through layer Ia of the olfactory cortex, where the dendrites were putatively contacted by axons of M/TCs (Fig. 5). Dendritic spines of VON neurons were apposed to immunoreactive VGluT1 elements in the axons of M/TCs (Fig. 6). In addition to morphological analysis using retrograde AAV encoding EGFP and tdTomato-expressing transgenic mice, we used a modified rabies virus to demonstrate that M/TC axons formed synaptic contacts with VON neurons (Fig. 7). Taking advantage of the neuronal pathway-specific infection of the rabies virus combining retrograde Cre viral vectors and Cre-dependent expression of viral receptors and glycoproteins^27^, we successfully infected VON neurons with the modified rabies virus. We observed that M/TCs in the OB were labelled by this retrograde trans-synaptic method. These results confirmed axonal inputs from M/TCs and suggested that the VON meets the criterion of the olfactory cortex.

The disynaptic pathway from the OB to the LH via the VON seems to be a shortcut pathway that conveys odorant information to the LH, bypassing other olfactory cortical regions. The efficacy of our rabies virus mediated-labelling of presynaptic M/TCs was insufficient to determine whether topographic axonal projections from the OB to the VON existed (Fig. 7c,d), as for other subregions of the olfactory cortex^32^. It remains to be addressed what odorant information is conveyed by VON GABAergic outputs to the LH. In addition to the OB, the VON receives inputs from other regions of the olfactory cortex including the AON, APC, OT, and PPC; modulatory inputs from the horizontal limb of the diagonal band; and top-down inputs from the prefrontal cortex (Fig. 7e,f). This organization suggests that VON neurons integrate these inputs and send GABAergic inhibitory output to the LH. Further studies should address the roles of each discrete input to VON neurons and how VON neurons integrate these inputs.

Rabies virus-mediated trans-synaptic labelling from the LH suggested that VON neurons formed synaptic contacts in the LH (Fig. 4). The LH consists of a variety of neuronal subtypes including orexin neurons, MCH neurons, and GABAergic neurons, which have distinct roles in eating and sleep/wakefulness^9,10,33^. It was recently reported that medium spiny neurons in the NAc expressing dopamine receptor D1 provide synaptic inputs to GABAergic neurons in the LH^20^. This NAc to LH pathway is involved in downregulation of feeding behaviour. Because our analysis did not identify discrete neuronal subtypes in the LH that received GABAergic inputs from the VON, further neuroanatomical studies should address this point. The current study only provides anatomical data on GABAergic neurons and their axonal projections to the LH, which does not necessarily prove the existence of functional connections between the VON and LH. In addition, we did not test the possibility that VON neurons send axonal projections to regions other than the LH. The possibility remains that the VON is part of a neuromodulatory region including GABAergic neurons such as the diagonal band^34^. Further functional assays using electrophysiology and optogenetics should address whether and how the VON influences the LH, as well as other potential target brain regions. Our findings on the VON provide novel insight into the circuitry that may underpin odour-induced behaviours.

## Materials and Methods

### Animals

All experiments were conducted in accordance with the Guidelines for Animal Experimentation in Neuroscience of the Japan Neuroscience Society and were approved by the Experimental Animal Research Committee of University of Fukui and Doshisha University. C57BL/6J male mice were purchased from Japan SLC. Homozygote Pcdh21-nCre mice (C57BL/6Cr-Tg(Pcdh21-cre)BYoko RBRC02189, RIKEN BRC)^23^ and homozygote Ai14 mice (B6;129S6-Gt(ROSA)26Sortm14(CAG-tdTomato)Hze/J 007908, The Jackson Laboratory)^22^ were crossed. Male heterozygote mice for both genes were used for experiments in Figs 4 and 5. All animals were individually housed after surgery on a 12/12 hour light/dark cycle. Food and water were available *ad libitum*.

### Virus preparation

For AAV vectors, AAVrg-CAG-GFP was a gift from Edward Boyden (Addgene viral prep #37825-AAVrg). AAV2-CMV-GFPCre, AAV2-CAG-Flex-TVA-mCherry, and AAV2-CAG-Flex-rabies G were packaged and concentrated to titres of 5.0 × 10^13^, 3.3 × 10^12^, and 1.3 × 10^12^ viral genomes/mL as previously reported^35^ using Addgene plasmids AAV-GFP/Cre (gift from Fred Gage, #49056^36^), CAG-Flex-TCB (gift from Liqun Luo, #48332^17^), and pAAV-CAG-FLEX-oG-WPRE-SV40pA (gift from Edward Callaway, #74292^16^), respectively.

For the rabies virus, we obtained EnvA-pseudotyped glycoprotein-deleted rabies virus encoding EGFP (SAD-dG-EGFP + EnvA) from Gene Transfer, Targeting and Therapeutics Facility of Salk Institute for Biological Studies at a titre of 2.9 × 10^7^ TU/mL.

For the lentivirus, NeuRet-Cre was packaged and concentrated to a titre of 1.1 × 10^12^ copies/mL as previously reported^35^.

### Stereotaxic surgery

Stereotaxic surgeries were performed on mice aged 10–16 weeks. Mice were anesthetized with a mixture of three anaesthetics (0.75 mg/kg medetomidine, 4 mg/kg midazolam, and 5 mg/kg butorphanol)^37^ and placed in a stereotaxic apparatus (Narishige, SR-5M). The skull above the targeted areas was thinned with a dental drill and carefully removed. Injections were conducted with a syringe pump (WPI, UltraMicroPump III) connected to a Hamilton syringe (Hamilton, RN-1701) and mounted glass micropipette with a tip diameter of 50 μm connected by an adaptor (Hamilton, 55750-01).

For data in Figs 1–3, we unilaterally injected 150 nL of CTB conjugated with Alexa 555 into the left LH using the following coordinates: A/P, −1.2 mm and M/L, 1.2 mm from bregma; D/V, 4.8 mm from the brain surface. One week later, the mice were deeply anaesthetised and fixed as described below.

For data in Fig. 4, we unilaterally injected 300 nL of 1:1:1 mixture of three AAVs (AAV2-CMV-GFPCre, AAV2-CAG-Flex-TVA-mCherry, and AAV2-CAG-Flex-rabies G) for rabies G(+) mice or 300 nL of 1:1 mixture of two AAVs (AAV2-CMV-GFPCre and AAV2-CAG-Flex-TVA-mCherry) for rabies G(−) mice into the left LH using the following coordinates: A/P, −1.2 mm and M/L, 1.2 mm from bregma; D/V, 4.8 mm from the brain surface. Two weeks later, we unilaterally injected 300 nL of SAD-dG-EGFP + EnvA using the same LH coordinates. One week later, the mice were deeply anaesthetised and fixed as described below.

For data in Figs 5 and 6, we unilaterally injected 300 nL of AAVrg-CAG-EGFP into the left LH of double transgenic mice using the following coordinates: A/P, −1.2 mm and M/L, 1.2 mm from bregma; DV, 4.8 mm from the brain surface. Two weeks later, the mice were deeply anesthetized and fixed as described below.

For data in Fig. 7, we unilaterally injected 300 nL of NeuRet-Cre into the left LH and 300 nL of 1:1 mixture of two AAVs (AAV2-CAG-Flex-TVA-mCherry and AAV2-CAG-Flex-rabies G) into the left VON. For the LH, we used the following coordinates: A/P, −1.2 mm and M/L, 1.2 mm from bregma; DV, 4.8 mm from the brain surface. For the VON, we used the following coordinates: A/P, +2.0 mm and M/L, 0.8 mm from bregma; DV, 4.2 mm from the brain surface. Two weeks later, we unilaterally injected 300 nL of SAD-dG-EGFP + EnvA using the same VON coordinates. One week later, the mice were deeply anaesthetised and fixed as described below.

### Sample preparation for histochemistry

Mice were deeply anaesthetised by intraperitoneal injection of sodium pentobarbital (150 mg/kg). They were transcardially perfused with phosphate-buffered saline (PBS) followed by 4% paraformaldehyde (PFA). The brains were removed from the skull, immersed in 4% PFA in 0.1 M phosphate buffer (PB) overnight, and then transferred to 30% sucrose in 0.1 M PB. The brains were then embedded in O.C.T. compound (Sakura Finetechnical), frozen at −80 °C, and sliced into coronal sections at a thickness of 20 μm with a cryotome. Sections were rinsed in PBS and 0.1 M PB, mounted on glass slides (Matsunami, CREST) using a paint brush, dried overnight in a vacuum desiccator, and then stored at 4 °C until histochemistry.

### Histochemistry

We performed immunostaining for orexin (Fig. 1a, middle), MCH (Fig. 1a, right), DARPP-32 (Figs 3, 4 and 7), EGFP (Figs 5 and 6), and VGluT1 (Fig. 6) as follows. The dried sections were rehydrated in PBS, permeabilised in TNT (0.1 M Tris-HCl; pH, 7.5; 0.15 M NaCl; 0.1% Tween 20), and blocked with 10% normal donkey serum diluted in TNT. Then, the sections were incubated with the following primary antibodies overnight at 4 °C: goat anti-orexin polyclonal antibody (1:400; Santa Cruz sc-8070); rabbit anti-MCH polyclonal antibody (1:400, Sigma M8440); rabbit anti-DARPP-32 monoclonal antibody (1:400; Abcam ab40801); rat anti-EGFP monoclonal antibody (1:1000, Nacalai Tesque 04404-84); and/or guinea pig anti-VGluT1 polyclonal antibody (1:500, Merck AB5905). After three washes in TNT, sections were incubated with the appropriate fluorescent dye-conjugated secondary antibodies (1:400; Jackson ImmunoResearch) for 2 hours at room temperature. After three washes in TNT, the sections were then counterstained with DAPI diluted in PBS (2 µg/mL) for 5 min. After washing in PBS, the sections were mounted in PermaFluor (Thermo Fisher Scientific).

For Fig. 2a, we performed *in situ* hybridization for *VGluT1*, *VGluT2*, *VGluT3*, and *GAD65/67* mRNA as follows. Digoxigenin (DIG)-labelled RNA probes were made using an *in vitro* transcription kit (Roche) according to the manufacturer’s protocol with plasmids kindly provided by Drs. Katsuhiko Ono and Yuchio Yanagawa^38–40^. The dried sections were fixed in 4% PFA, digested with Proteinase K (10 μg/mL) for 30 min, and post-fixed in 4% PFA. After prehybridization, the sections were incubated overnight at 65 °C with DIG-labelled RNA probes. After stringent washing, the sections were blocked with 10% normal sheep serum, 1% bovine serum albumin (BSA), and 0.1% Triton X-100 in PBS. Subsequently, the sections were incubated overnight at 4 °C with alkaline phosphatase-conjugated anti-DIG antibody (1:1000; Roche). The sections were washed in TNT, followed by alkaline phosphatase buffer (100 mM NaCl; 100 mM Tris-HCl; pH, 9.5; 50 mM MgCl_2_; 0.1% Tween 20; 5 mM levamisole). The sections were treated overnight with NBT/BCIP (Roche) mixture at room temperature in a dark room for colour development. Sections were subsequently rinsed in PBS and mounted in PermaFluor (Thermo Fisher Scientific).

For data in Fig. 2b, we performed fluorescent double labelling for CTB immunoreactivity and *VGluT1* or *GAD65/67* mRNA as follows. Fluorescein-labelled RNA probes were prepared as described above. Hybridization and washing were performed as described above, except that fluorescein-labelled probes were used for hybridization. After blocking in 1% blocking buffer (11096176001, Roche) for 1 h, the fluorescent-labelled probes were detected. The sections were incubated with an anti-fluorescein antibody conjugated with horseradish peroxidase (1:500; Perkin-Elmer) for 1 h at room temperature. After three 10-min washes in TNT, the sections were treated with diluted (1:100) TSA-Plus dinitrophenol (DNP) reagents for 5 min according to the manufacturer’s instructions (Perkin-Elmer), and the FLU signals were converted to DNP signals. To amplify the DNP signals, the sections were washed in TNT three times for 10 min each, incubated with an anti-DNP antibody conjugated with horseradish peroxidase (1:500; Perkin-Elmer) for 1 h at room temperature, and treated again with diluted TSA-Plus DNP reagents (1:100) for 5 min. Subsequently, the sections were incubated overnight with an anti-DNP antibody conjugated with Alexa 488 (1:500; Molecular Probes) in 1% blocking buffer at 4 °C for fluorescent detection of DNP signals. At this point, a goat anti-CTB antibody (1:500; List Biological Laboratories #703) was added to the incubation mixture for detection of CTB. The sections were washed three times in TNT and incubated with a Cy3-conjugated secondary antibody (1:400; Jackson ImmunoResearch Labs) for 2 h. After three washes in TNT, the sections were then counterstained with DAPI diluted in PBS (2 µg/mL) for 5 min. After washing in PBS, the sections were mounted in PermaFluor (Thermo Fisher Scientific).

For data in Figs 1a (left panel), 1b, 2a (two left panels), and 7, the dried sections were rehydrated in PBS and counterstained with DAPI diluted in PBS (2 µg/mL) for 5 min. After washing in PBS, the sections were mounted in PermaFluor (Thermo Fisher Scientific).

### Microscopy

Sections were examined with a bright field virtual slide system (Hamamatsu Photonics, NanoZoomer), a fluorescent microscope (Olympus, BX51WI), and a confocal laser microscope (Olympus, FV1200).

### Data sampling and statistics

For the CTB-Alexa 555 injections into the LH (Figs 1–3 and Supplementary Figs 1–3), 10 mice were used. All mice showed CTB-labelled cells in the VON. Four mice that satisfied the criteria for successful injection, whereby tracer did not spread to the thalamus, amygdala, or other hypothalamic areas, were selected for image acquisition and quantitative analysis. The minimal spread of CTB-Alexa 555 in the LH which resulted in substantial labelling in the VON is shown in Supplementary Fig. 1. For quantification of the percentage of marker mRNA(+) cells among CTB-labelled cells in Fig. 2 and Supplementary Fig. 2, we used three coronal sections containing the VON and medial prefrontal cortex per mouse. Soma size of CTB-labelled cells in Fig. 2d was measured by delineating an outline of CTB-labelled soma using Image J and represented as μm^2^.

For modified rabies-mediated transsynaptic tracing from the VON (Fig. 4), we performed injections in nine mice and selected three mice for quantitative analysis that exhibited localization of starter cells in the LH with no distribution in other areas (thalamus, amygdala or other hypothalamic areas). The number of sections used for quantifying EGFP(+) cells in the VON ranged from 7–8 coronal sections per mouse.

For AAVrg-CAG-EGFP injections into the LH of transgenic mice (Figs 5–6 and Supplementary Fig. 4), seven mice were used. All mice showed EGFP-labelled cells in the VON. Three mice (two mice for coronal sections and one mouse for sagittal sections) that satisfied the criteria that EGFP expression in the injection site (LH) did not spread to the thalamus, amygdala, or other hypothalamic areas were selected for image acquisition and analysis. For data in Fig. 5, we selected five sections and examined dendrites of VON neurons (EGFP), axons of M/TCs (tdTomato), and DARPP-32 expression. For data in Fig. 6, we selected three 20-μm-thick sections including axonal innervation of M/TCs to dendrites of VON neurons. We confirmed more than one putative synaptic contact (Fig. 6) in all nine sections using colocalization analysis with XZ-YZ views (60x lens with 2x digital zoom, 0.5 μm-z step).

For modified rabies-mediated transsynaptic tracing from the VON (Fig. 7), we performed injections in 11 mice and selected three mice for quantitative analysis that exhibited localization of starter cells in the VON with no distribution in other olfactory cortical areas.

The structure boundaries in Figures were drawn using the Franklin and Paxinos mouse brain atlas^31^. Data are shown as average and SD with individual plots (except Fig. 2d, without individual plots). Statistical differences were tested using parametric tests (unpaired *t*-test, Fig. 2c,d and Supplementary Fig. 2b; one-way ANOVA with post-hoc Tukey’s test, Figs 1c and 4e; two-way ANOVA with post-hoc Tukey’s test, Fig. 4c) using GraphPad Prism 7.

## Supplementary information

Supplementary Information

## Data Availability

Any data associated with the study is available from the authors upon request.
