# Supplementary material for: GABAergic neurons in the olfactory cortex projecting to the lateral hypothalamus in mice
Source: Sci Rep. 2019 May 9;9:7132. doi: 10.1038/s41598-019-43580-1 (PMC6509143; doi:10.1038/s41598-019-43580-1)
Supplement: Supplementary file 1 — Supplementary Information [file 41598_2019_43580_MOESM1_ESM.pdf]

Scientific Reports

Supplementary Information

GABAergic neurons in the olfactory cortex projecting to the lateral hypothalamus in mice

Koshi Murata, Tomoki Kinoshita, Yugo Fukazawa, Kenta Kobayashi, Kazuto Kobayashi, Kazunari Miyamichi, Hiroyuki Okuno, Haruhiko Bito, Yoshio Sakurai, Masahiro Yamaguchi, Kensaku Mori, and Hiroyuki Manabe

Inventory of Supplementary Information

Supplementary Figure 1

Supplementary Figure 2

Supplementary Figure 3

Supplementary Figure 4

Supplementary Figure 1

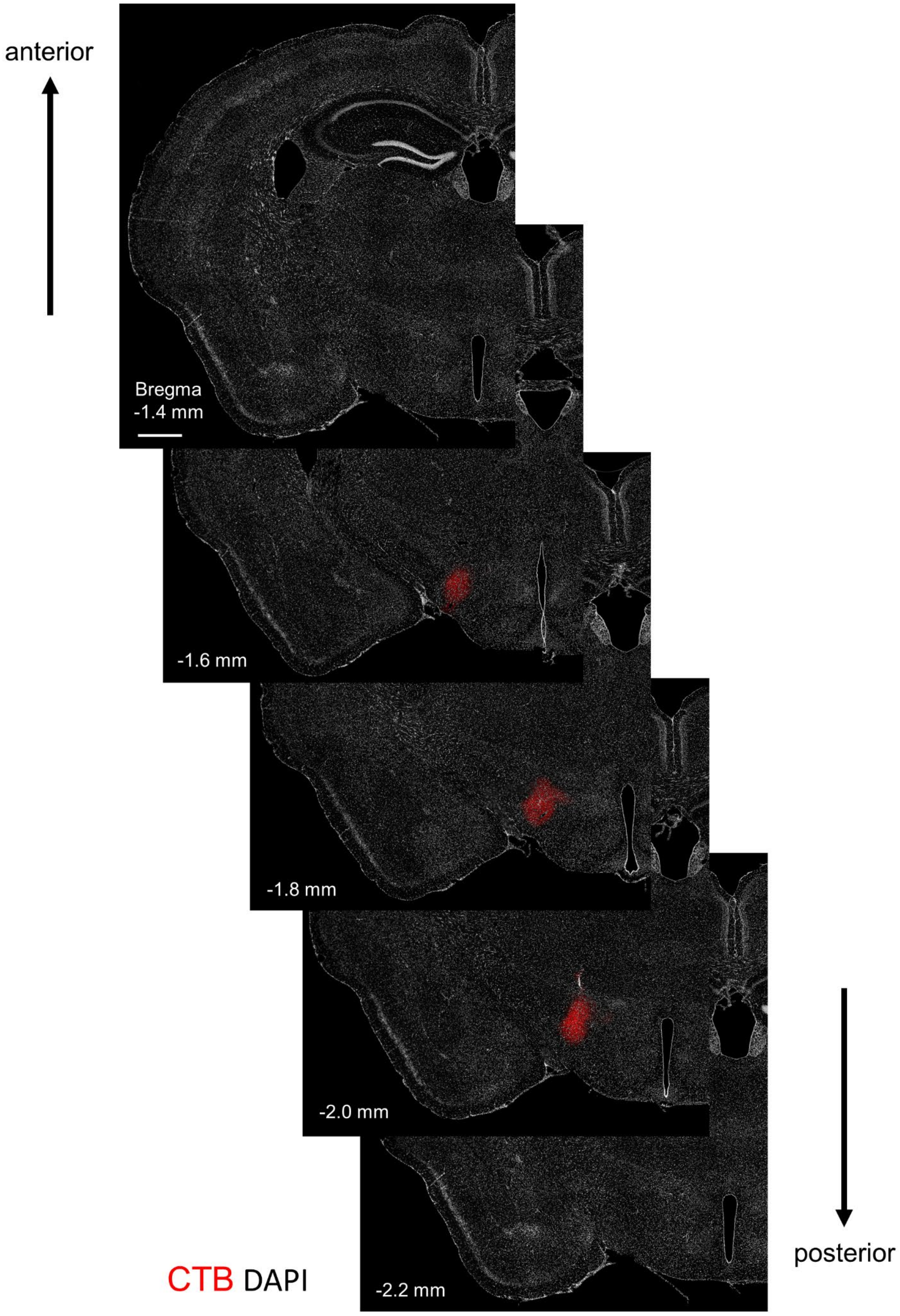

Supplementary Figure 1 Spread of CTB-Alexa 555 in the LH

Coronal sections including the injection site of CTB-Alexa 555 into the LH. Sections are arranged at 200 μm intervals in the antero-posterior axis. Scale bar: 500 μm.

Supplementary Figure 2

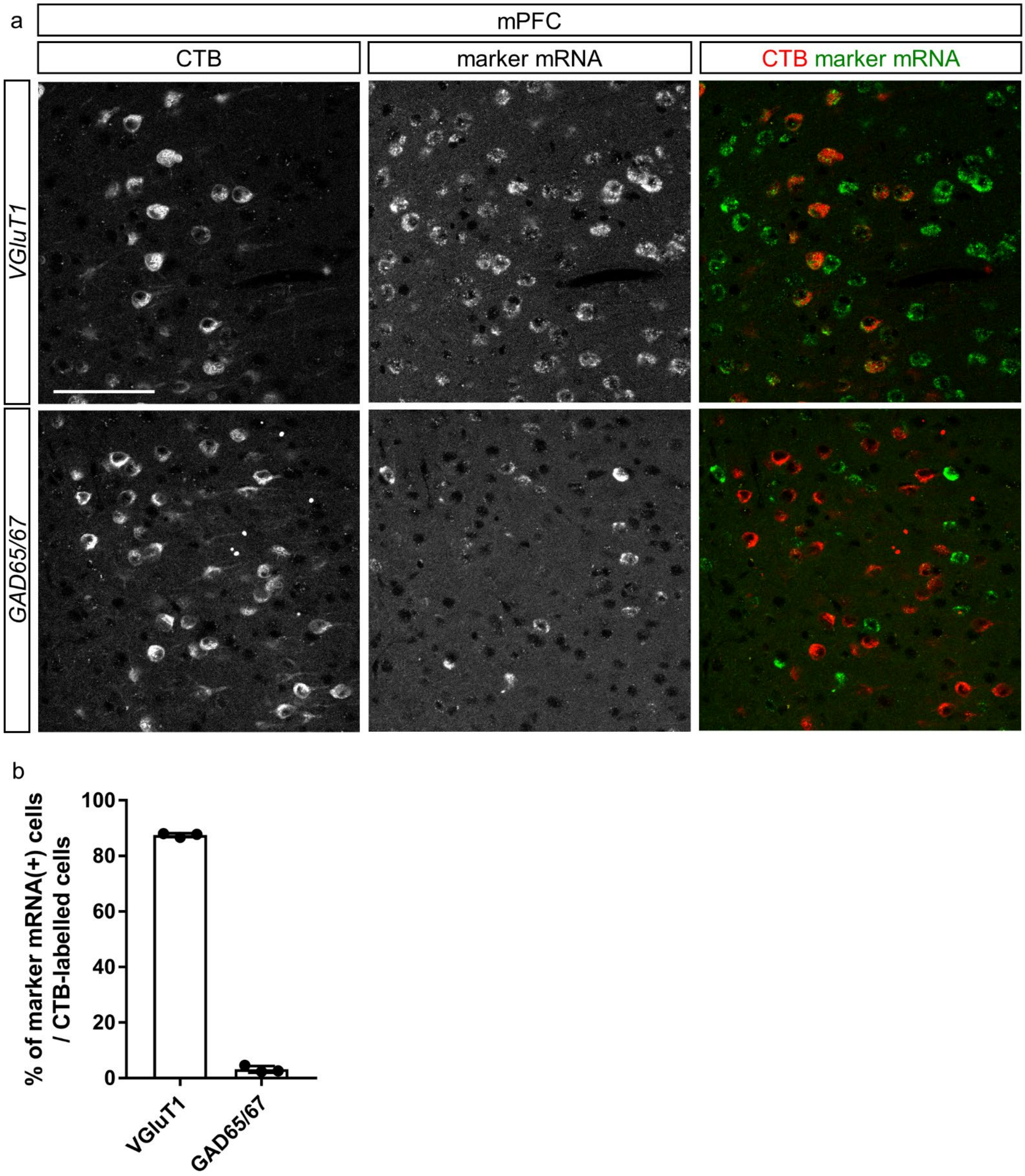

Supplementary Figure 2 Specificity of fluorescent *in situ* hybridization for *VGluT1* and *GAD65/67* mRNAs

(a) Double fluorescent labelling for CTB and *VGluT1* (upper panels) or *GAD65/67* (lower panels) mRNAs in the medial prefrontal cortex (mPFC). Scale bar: 100 μm. (b) Percentage of *VGluT1* or *GAD65/67* mRNA(+) cells among CTB-labelled cells in the mPFC. Data are shown as average ± SD with individual data plots.

Supplementary Figure 3

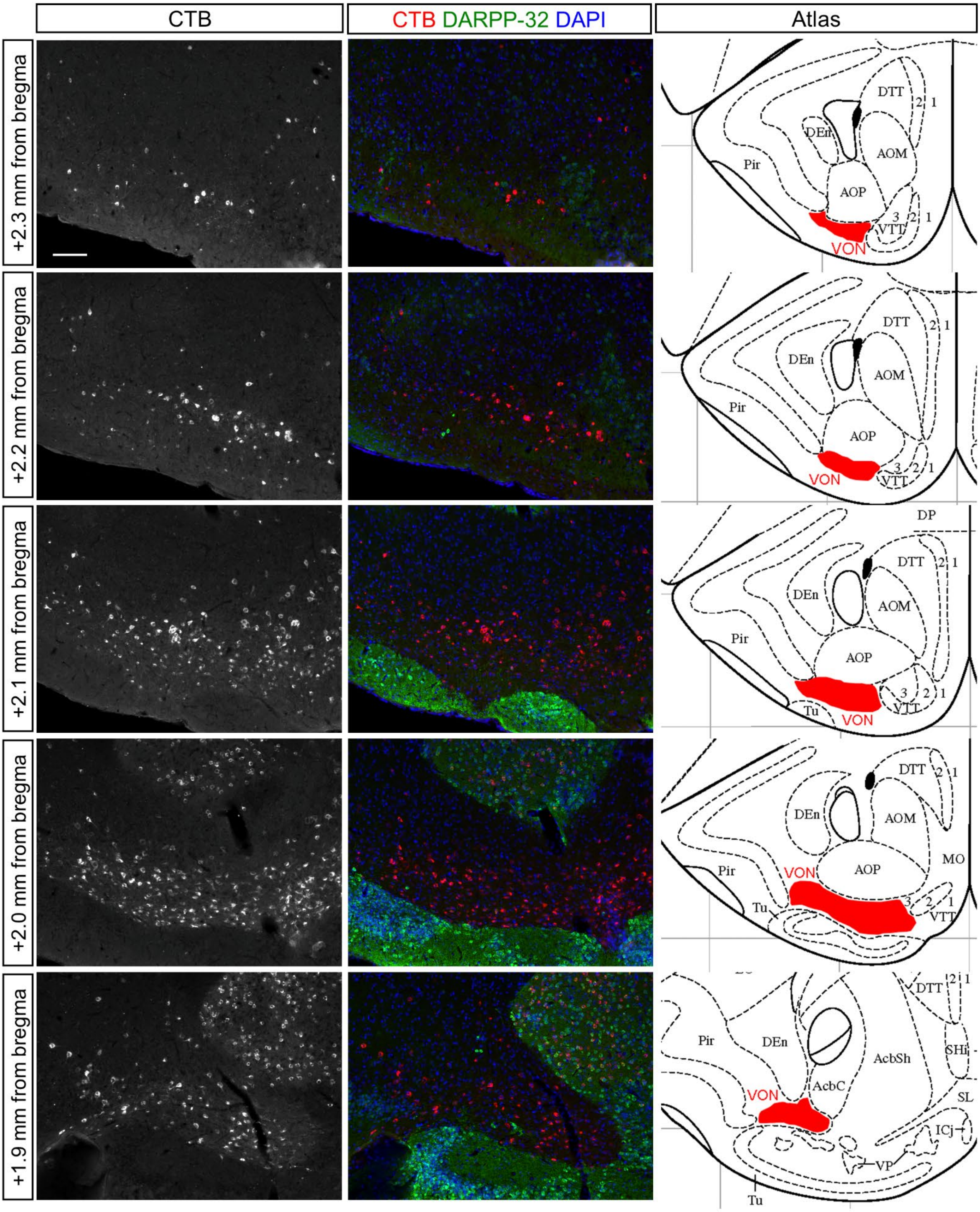

Supplementary Figure 3 Distribution of the VON in coronal sections

Coronal sections of the VON after injection of CTB-Alexa 555 (left panels and red in middle panels) into the LH with immunostaining for DARPP-32 (green) and DAPI staining (blue). Right panels were adopted from Franklin and Paxinos's mouse brain atlas[31]. Pir, anterior piriform cortex; DEn, dorsal endopiriform nucleus; DTT, dorsal tenia tecta; AOM, anterior olfactory nucleus medial part; AOP, anterior olfactory nucleus posterior part, VTT, ventral tenia tecta; VON, ventral olfactory nucleus; Tu, olfactory tubercle; MO, medial orbital cortex; SHi, septohippocampal nucleus; SL, semilunar nucleus; AcbSh, nucleus accumbens shell, AcbC, nucleus accumbens core. Scale bar: 100 μm.

Supplementary Figure 4

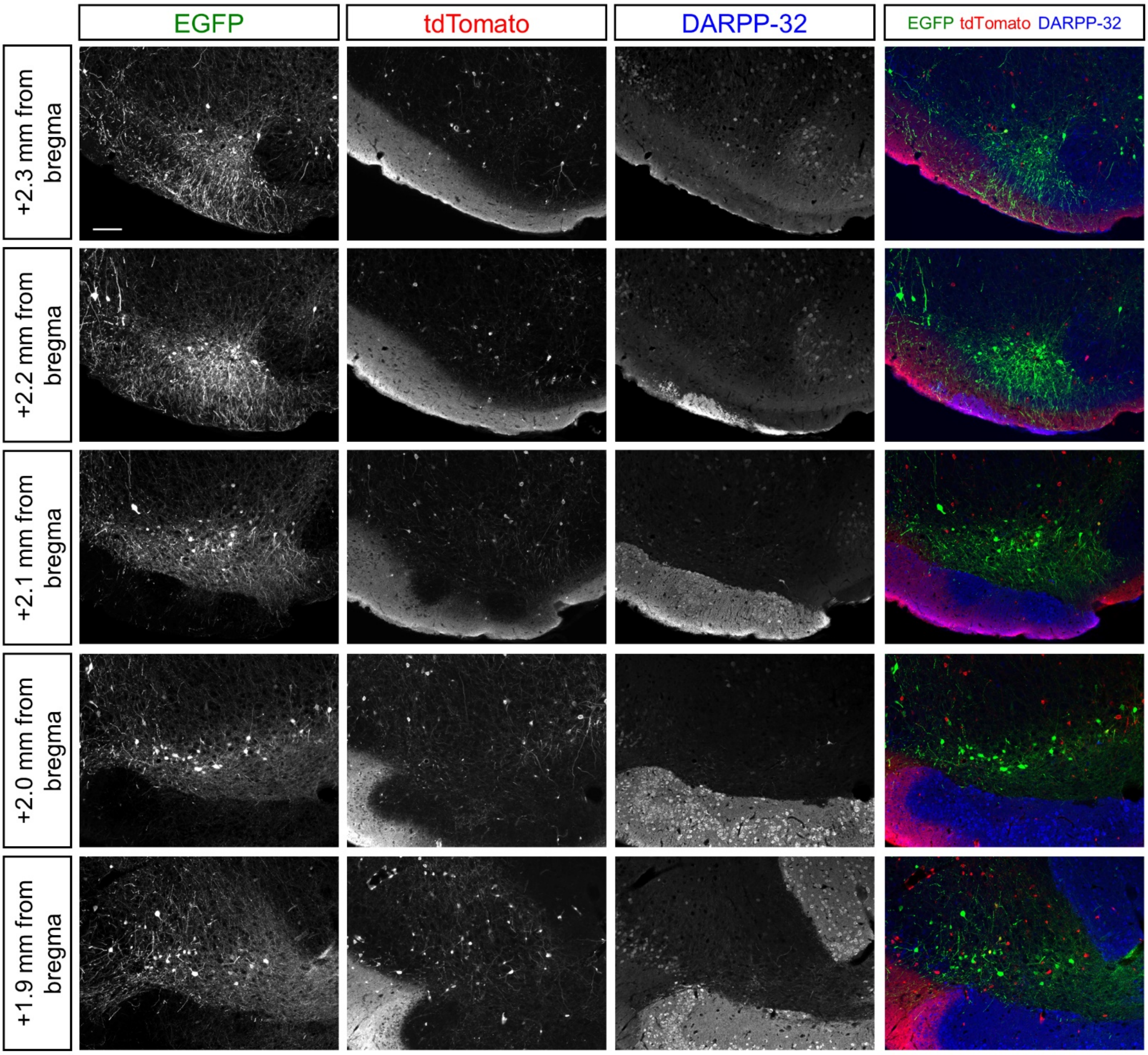

Supplementary Figure 4 Axonal innervation from mitral and tufted cells in the olfactory bulb to the VON

Coronal sections of the VON including EGFP-labelled cells (left panels and green in right panels) in a tdTomato-labelled transgenic mouse (second panels from left column and red in right panels) after injection of retrograde AAV vector encoding EGFP into the LH with immunostaining for DARPP-32 (second panels from right column and blue in right panels). Dendrites of EGFP-labelled VON neurons (green) innervated layer Ia of the olfactory cortex (red). Scale bar: 100 µm.
